# Recruitment into diabetes prevention programs: what is the impact of errors in self-reported measures of obesity?

**DOI:** 10.1186/1471-2458-12-510

**Published:** 2012-07-08

**Authors:** Andrea Hernan, Benjamin Philpot, Edward D Janus, James A Dunbar

**Affiliations:** 1 Greater Green Triangle University Department of Rural Health, Flinders University and Deakin University, PO Box 423, Warrnambool, Victoria, 3280, Australia; 2 Department of Medicine, University of Melbourne, Western Hospital, Gordon Street, Footscray, Victoria, 3011, Australia

**Keywords:** Type 2 diabetes, Prevention programs, Recruitment, Weight misperception, BMI, Waist circumference

## Abstract

**Background:**

Error in self-reported measures of obesity has been frequently described, but the effect of self-reported error on recruitment into diabetes prevention programs is not well established. The aim of this study was to examine the effect of using self-reported obesity data from the Finnish diabetes risk score (FINDRISC) on recruitment into the Greater Green Triangle Diabetes Prevention Project (GGT DPP).

**Methods:**

The GGT DPP was a structured group-based lifestyle modification program delivered in primary health care settings in South-Eastern Australia. Between 2004–05, 850 FINDRISC forms were collected during recruitment for the GGT DPP. Eligible individuals, at moderate to high risk of developing diabetes, were invited to undertake baseline tests, including anthropometric measurements performed by specially trained nurses. In addition to errors in calculating total risk scores, accuracy of self-reported data (height, weight, waist circumference (WC) and Body Mass Index (BMI)) from FINDRISCs was compared with baseline data, with impact on participation eligibility presented.

**Results:**

Overall, calculation errors impacted on eligibility in 18 cases (2.1%). Of n = 279 GGT DPP participants with measured data, errors (total score calculation, BMI or WC) in self-report were found in n = 90 (32.3%). These errors were equally likely to result in under- or over-reported risk. Under-reporting was more common in those reporting lower risk scores (Spearman-rho = −0.226, *p*-value < 0.001). However, underestimation resulted in only 6% of individuals at high risk of diabetes being incorrectly categorised as moderate or low risk of diabetes.

**Conclusions:**

Overall FINDRISC was found to be an effective tool to screen and recruit participants at moderate to high risk of diabetes, accurately categorising levels of overweight and obesity using self-report data. The results could be generalisable to other diabetes prevention programs using screening tools which include self-reported levels of obesity.

## Background

Several clinical trials have demonstrated that lifestyle modification with weight loss and moderate physical exercise can reduce the incidence of type 2 diabetes by up to 58% for people at high risk [1]. The Greater Green Triangle Diabetes Prevention Project (GGT DPP) was a ‘real world’ implementation trial based on the Finnish Diabetes Prevention Study (DPS) [2] and the Good Ageing in Lahti Region (GOAL) Lifestyle Implementation Trial [3]. The GGT DPP was a structured group-based lifestyle modification program delivered in Australian primary health care settings from 2004–2006. Its aim was to prevent the onset of type 2 diabetes among moderate to high risk individuals utilising evidence based behaviour change theories [4].

Recruitment of people at high risk is a major part of diabetes prevention programs. One method for recruiting high risk individuals into these programs is through the use of a diabetes risk score. In the GGT DPP diabetes risk was ascertained using the Finnish diabetes risk score (FINDRISC) [5]. FINDRISC contains eight questions regarding risk factors for diabetes, and includes waist circumference and Body Mass Index (BMI, kg/m^2^) based on height and weight, which were all self-reported in the GGT DPP recruitment.

Epidemiological studies often use self-reported data to ascertain anthropometric information of a given population in order to estimate levels of risk for chronic diseases, such as type 2 diabetes. One of the most important risk factors for type 2 diabetes is obesity [6]. Overweight and obesity estimates are generally determined from BMI [7]. Waist circumference and waist to hip ratio have also been used as indicators to estimate prevalence of overweight and obesity, particularly central adiposity [7,8].

BMI calculated from self-report height and weight, derived from telephone interview or questionnaire, is a low cost method to gather population data, but the validity and measurement error apparent in the use of these self-report data remains a contentious issue. There have been numerous small scale studies [9-11], the largest population based study comprising 21 789 individuals [12], and a systematic review [13] investigating the error between self-reported height and weight and BMI and actual measured height and weight, and BMI. These studies found that generally weight is underestimated and height is overestimated, and consequently BMI is underestimated. However, there are some studies that report the opposite effect [14,15]. A number of characteristics have been shown to be linked with incorrectly self-reporting weight and height and include; gender usually female, older age, ethnicity, presence of obesity, lower socio-economic status, presence of disability, and social desirability for thinness and tallness [11-13]. Errors between self-reported and measured waist circumference have also been reported in the literature, with under-reporting being the most consistent finding [12,16,17].

Errors in self-report data have implications for population health surveillance, obesity estimates, and resource allocation, and could impact upon recruitment into health prevention programs, such as type 2 diabetes prevention programs that target high risk individuals.

This study aims to examine the effect of error in self reported measures of obesity on recruiting into diabetes prevention programs, using data from the GGT DPP about the accuracy of self-reported compared with objectively measured height, weight, waist circumference and BMI.

## Methods

The methods and recruitment process have been previously described [4]. Briefly, this project was carried out across three sites in Southeast Australia. Recruitment into this study occurred through opportunistic screening by study nurses of individuals aged 40–75 years presenting at local general practices. FINDRISC was used to identify individuals at moderate to high risk of developing type 2 diabetes [5].

### FINDRISC

FINDRISC was developed in Finland from population based epidemiological studies and provides a total score that predicts future diabetes risk [5]. The tool is a one page form that contains eight questions with weighted categorical answers about age, BMI, waist circumference, physical activity, daily consumption of fruits, berries or vegetables, history of antihypertensive drug treatment, history of high blood glucose, and family history of diabetes. The total risk score is a sum of the individual scores from each of the eight questions, with totals ranging from zero to 26 [18].

During recruitment for the GGT DPP, potential participants were asked to fill in the anthropometric information on the FINDRISC from self-report. This included data for questions about height, weight and waist circumference category. Study nurses were available to provide assistance if necessary, but were not required to perform anthropometric measures. However, after contacting the study nurses from all sites, one site was excluded from the analysis between self-report and measured data as the study nurse measured height, weight, and waist circumference during recruitment.

The study nurse was required to calculate BMI from self-reported height (m) and weight (kg), and then the total risk score. They were provided with a handheld BMI calculator to determine BMI and then record an individual’s BMI category. The BMI calculator has measurement markings for height between 1.4 and 2.1 m with 1 cm increments, weight between 40 and 120 kg with 1 kg increments, and BMI between 10 and 60 kg/m^2^ with 1 kg/m^2^ increments. Conversion charts for height from feet and inches (5’ 0” to 6’ 6”) to cm and weight from stone (8 to 26) to kg were also provided (all to 0 decimal places).

BMI categories were based on the World Health Organisation’s International Classifications, with normal BMI (<25.0 kg/m^2^) scored ‘0’, overweight (BMI 25.0-29.9 kg/m^2^) scored ‘1’, and obese (BMI ≥30.0 kg/m^2^) scored ‘3’ [19].

Waist circumference categories were different for men and women based on Lean’s (1995) ‘waist action levels’ that correspond to BMI categories of normal, overweight and obese [20]. For both men and women normal categories (men <94.0 cm, women <80.0 cm) were scored ‘0’, overweight categories (men 94.0 cm-101.9 cm, women 80.0 cm-87.9 cm) were scored ‘3’, and obese categories (men ≥102.0 cm, women ≥88.0 cm) were scored ‘4’.

The recruitment criteria for the GGT DPP included a total score of 12 or more on the FINDRISC (moderate to high risk). This score was selected to be comparable with the GOAL Lifestyle Implementation Trial recruitment criteria [3]. Individuals with cancer, recent myocardial infarction or stroke, cognitive impairment, substance abuse, pregnancy or previous type 2 diabetes diagnosis were excluded from the study [4].

Individuals recruited to the study attended baseline clinical tests within three weeks of being screened with the FINDRISC. Objectively measured height, weight, and waist circumference were among the anthropometric measures taken by specially trained study nurses using the European Health Risk Monitoring protocol [21]. Specifically, waist circumference was measured with a non-stretched plastic tailor's measuring tape. Participants were measured at a level midway between the lower rib margin and the iliac crest with a tape around the body in a horizontal position with their clothes removed. Participants stood normally with their feet approximately 12–15 cm apart, so their weight should be equally distributed between each leg. Additionally, participants were asked to breathe normally with the reading of the measurement being taken at the end of gently exhaling.

Height and waist circumference were measured in cm to 1 decimal place, and weight was measured in kg to 1 decimal place. For waist circumference, the mean of two measurements was used.

### Analysis

Data were analysed in PASW Statistics (SPSS) version 18. Absolute numbers with percentages are presented in the results section.

After considering the time between recruitment and baseline clinical testing, daily fluctuation of weight [22], and potential waist circumference measurement error, borderline categories were created for BMI and waist circumference. The two extra categories of BMI were ‘borderline normal/overweight’ (borderline N/OW, 25 kg/m^2^ ± 2 kg) and ‘borderline overweight/obese’ (borderline OW/OB, 30 kg/m^2^ ± 2 kg). A 2 kg allowance has been previously used by Rossouw et al. (2000) in determining the accuracy of self-reported weight in overweight or obese subjects [22]. Similarly, borderline categories were created for waist circumference that allow for a ± 2 cm margin of error.

Paired T-test was used to determine the difference between self-reported and measured BMI. Spearman-rho was used to calculate the correlation between original risk score total and risk score error in those who attended baseline clinical testing.

### Ethics

This study was approved by the Flinders University Clinical Research Committee (reference number 105/034). Individuals who participated in the GGT DPP (n = 347) provided written and informed consent.

## Results

**Figure 1:**
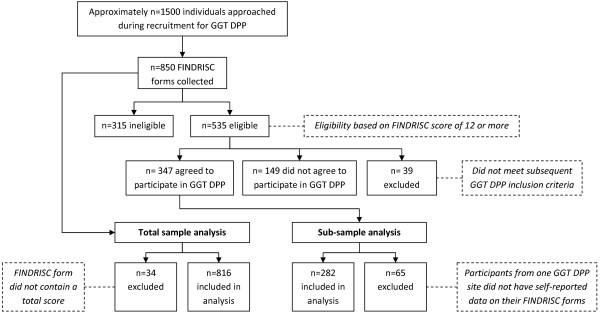
Flow chart of information collected during GGT DPP recruitment and inclusion/exclusion criteria for analysis.

Approximately 1,500 people were approached to undertake the FINDRISC, with a total of n = 850 FINDRISC forms being collected during recruitment for the GGT DPP. The total (n = 850) represents individuals (63% female) who were ineligible (n = 315), excluded (n = 39), eligible but did not participate (n = 149), and those eligible who agreed to participate (n = 347) (Figure 1).

The mean self-reported height and weight for men were 176.4 cm (SD 6.5) and 93.0 kg (SD 16.5) respectively and for women 163.0 cm (SD 6.9) and 82.3 kg (SD 17.5).

### Total sample (calculation errors in self-report)

A total of n = 816 FINDRISC forms contained a total score. Mean FINDRISC total score was 12.8, (SD = 4.6). Errors in either BMI calculations or total score calculations or both were found in 128 forms. For total scores, n = 88 (10.8%) were calculated incorrectly by the study nurse or potential participant, with 36 having under-reported risk and 52 having over-reported risk. There were some differences between BMI categories selected on the risk score versus BMI calculated correctly from height and weight on the risk score. BMI category errors resulted in 37 individuals (4.4%) over-reporting their BMI and 16 (1.9%) under-reporting their BMI, while 90.1% (n = 766) were correct, and 3.6% (n = 31) had missing data.

These errors were assessed to determine their impact on eligibility for participation in the GGT DPP. Errors impacted on eligibility in 18 cases (2.1%). Twelve who were originally eligible (initial scores 12, 13, 14) were actually ineligible (recalculated scores 9, 10, 11). Six who were originally ineligible (initial scores 7, 10, 11) were actually eligible (recalculated scores 12 and 13).

### Sub-sample (actual vs. self-report)

There were a total of n = 347 who were eligible for analysis, however, participants from one site (n = 65) were excluded, as self-report data were not available due to the nurse measuring height, weight, and waist circumference.

BMI category reported on the FINDRISC was compared with BMI calculated from measured data for those who were eligible and participated. A total of n = 278 had full information available for this comparison. Based on BMI category, n = 33 (11.9%) had under-reported and n = 13 (4.7%) had over-reported BMI. After allowing for a ± 2 kg margin of error, n = 23 (8.3%) had under-reported and n = 7 (2.5%) had over-reported BMI category (Table 1). Mean self-reported BMI was lower than measured BMI (32.7 vs. 33.8, *p* < 0.001). A total of n = 200 (71.9%) individuals had under-reported BMI to some extent.

**Table 1:** Self-reported BMI category vs BMI category as calculated from measured data

	**Self-reported BMI category**			
**Actual BMI category**	**Normal(n)**	**Overweight (n)**	**Obese (n)**	**Total**
Normal	2	**1**^**¤**^	**0**^**¤**^	3
Borderline N/OW*	1	6	**0**^**¤**^	7
Overweight	**4**^**§**^	40	**6**^**¤**^	50
Borderline OW/OB*	**0**^**§**^	20	6	26
Obese	**0**^**§**^	**19**^**§**^	173	192
**Total**	7	86	185	278

*Categories represent those within 2 kg of cutoffs for normal and overweight (N/OW), and overweight and obese (OW/OB).

Notes for table: § represents participants who under-report their BMI and ¤ represents those who over-report their BMI, after allowing for 2 kg margins of error.

Self-report and actual waist circumference category errors were calculated similarly to BMI above. Based on waist circumference category for both men and women (n = 281), 40 (14.2%) under-reported and 8 (2.8%) over-reported their waist circumference. After allowing for a ± 2 cm margin of error, 27 (9.6%) under-reported and 4 (1.4%) over-reported their waist circumference category (Table 2).

**Table 2:** Self-reported Waist Circumference category vs measured Waist Circumference category

	**Self-reported Waist Circumference category**			
**Actual Waist Circumference category**	**Normal (n)**	**Overweight (n)**	**Obese (n)**	**Total**
Normal	2	**2**^**¤**^	**0**^**¤**^	4
Borderline N/OW*	2	5	**0**^**¤**^	7
Overweight	**0**^§^	7	**2**^**¤**^	9
Borderline OW/OB*	**0**^§^	16	9	25
Obese	**2**^§^	**25**^§^	209	236
**Total**	6	55	220	281

* Categories represent those within 2 cm of cutoffs for normal and overweight (N/OW), and overweight and obese (OW/OB).

Notes for table: § represents participants who under-report their waist circumference category and ¤ represents those who over-report their waist circumference category, after allowing for 2 cm margins of error.

The level of under-reporting, correct reporting and over-reporting for both BMI and waist circumference categories after allowing for margins of error can be viewed in Table 3. In total, 58 (21.0%) over- or under-reported BMI or waist circumference category. Under-report was most common.

**Table 3:** Under-report and Over-report for Waist Circumference and/or BMI (allowing for error margin*)

	**Waist Circumference Status**			
**BMI Status**	**Under-report (n)**	**Correct (n)**	**Over-report (n)**	**Total**
Under-report	**1**^**#**^	**21**^**#**^	**1**^**#**^	23
Correct	**25**^**#**^	219	**3**^**#**^	247
Over-report	**1**^**#**^	**6**^**#**^	**0**^**#**^	7
**Total**	27	246	4	277

*Under-report and Over-report categories include allowances for 2 kg and 2 cm margins of error.

Notes for table: # represents participants who either under-reported or over-reported at least one of BMI or waist circumference.

Table 4 shows the corrected risk score and the over-reporting or under-reporting errors in this risk score. Total risk scores were calculated correctly for 189 (68.7%, Table 4). However, risk scores from self-report were over-reported for 39 participants (14.0%), resulting in two participating in the program despite being ineligible based on anthropometric measurements. Self-reported risk scores were under-reported by 51 participants (18.3%).

**Table 4:** Corrected Risk Score vs Error in Risk Score*

**Categories of risk**	**Corrected Risk Score**	**Risk Error**										**Total individuals**
		**Over-report Risk**					**Correct**	**Under-report Risk**				
		*−5*	*−4*	*−3*	*−2*	*−1*	*0*	*1*	*2*	*3*	*4*	
Low	10			**1**^**¤**^								1
	11					**1**^**¤**^						1
Moderate	12				5	1	27					33
	13			**1**^**†**^	**3**^**†**^		15	3				22
	14			**2**^**†**^	**1**^**†**^		26	5	4			38
High	15				4	2	16	**3**^**#**^	**3**^**#**^	**1**^**#**^		29
	16			3	1	3	22	8	**1**^**#**^	**1**^**#**^	**1**^**#**^	40
	17	1			1	2	20	2	5			31
	18					1	20	2	4		**1**^**#**^	28
	19				1		14		2			17
	20						11	2	1	1		15
	21				1	2	6					9
	22				1		3					4
	23			1			5					6
	24						2				1	3
	25						2					2
	26											0
	**Total**	1	0	8	18	12	189	25	20	3	3	279

*Errors in risk score include allowances of 2 kg and 2 cm margins of error.

Notes for table: ¤ indicates participants who over-reported their risk and were consequently ineligible based on anthropometric measurements.

† indicates participants at high risk who were actually at moderate risk based on anthropometric measurements.

# indicates participants at moderate risk who were actually at high risk based on anthropometric measurements.

Under-reporting was generally more common in those reporting lower risk scores (Table 4), with a low correlation found between risk score and error (Spearman-rho = −0.226, *p*-value < 0.001).

Table 4 also demonstrates errors in self report by categories of risk. A total of 11 participants out of the 180 participants at high risk (6.1%) were incorrectly categorised as moderate risk.

## Discussion

We believe this is the first study describing the effect of error in self reported measures of obesity on recruitment into a diabetes prevention program. Errors in self-report were found in one in five participants in the GGT DPP, with over half of these resulting in underestimations of type 2 diabetes risk based on the FINDRISC tool. However, the impact of the underestimation only resulted in 6% of individuals at high risk of diabetes being incorrectly categorised as moderate or low risk of diabetes. Therefore FINDRISC was generally found to be an effective tool to screen and recruit participants at moderate to high risk of diabetes. This finding also suggests that self-reported levels of obesity may be adequate to use in other screening tools for the prevention of type 2 diabetes.

The accuracy of self-reported BMI and waist circumference found in this study are consistent with evidence suggesting that generally there is an overestimation of height, underestimation of weight, and therefore an underestimation of BMI [9-13]. This result has been found in populations who are overweight or obese [9,22], and in middle aged or elderly populations [23,24]. In this study, when using BMI as a continuous measure, this is particularly notable for the two thirds of individuals who under-reported their BMI to some extent. Waist circumference followed a similar trend with under-estimation being more common than over-estimation. To better approximate levels of obesity, other studies have adjusted self-reported data for a number of confounding factors [9,11,14]. This has been found to be a viable methodology for developing population estimates. For recruitment into diabetes prevention programs, accuracy in assessing risk at the individual level is required.

Central obesity, as measured by waist circumference, is an increasingly recognised important risk factor for type 2 diabetes. This is now well recognised by health professionals, but to a lesser extent by the general population [25]. Unlike weight, waist circumference is not commonly measured by individuals, and it has been shown that individuals may find it difficult to measure their waist circumference accurately [9]. Additionally, clothing sizes vary and can lead to misperception. Waist circumference misperception is demonstrated in this study with a larger proportion of participants inaccurately categorising waist circumference than height and weight. Since waist circumference is such an important risk factor for diabetes and cardio-metabolic diseases, more health promotion and social marketing is needed to increase public awareness [25].

Anthropometric information used to derive FINDRISC was determined from measured data [5], unlike the self-reported methods used to assess risk in the GGT DPP. Using self-reported weight, height, and waist circumference in the real world may reduce FINDRISC’s demonstrated high sensitivity, specificity and predictive value. But the results from this study have shown that the participants in this study were generally correctly identified by FINDRISC as being at moderate to high risk of diabetes. It is expected that the majority of those at risk of diabetes were captured, with only some being missed due to self-report errors.

There are some limitations in this paper that need to be taken into consideration, one of which includes the possibility of response bias. Potential participants and facilitators may have biased answers on purpose in order to show less or more risk. For example, individuals not wanting to participate may modify their responses to avoid recruitment. Conversely, in individuals who are perceived to be on the borderline for recruitment, study nurses might have prompted answers which took their score up to 12 and encouraged them to join the program. The time lag between self-report and clinical testing could affect the measurement comparisons. Since margins of error were included to account for this, the estimate of 6.1% of high risk individuals incorrectly categorised as moderate risk is a conservative estimate. The specific results of this study may not be generalisable as the study population were recruited opportunistically from general practices. Anthropometric measurements were not available for n = 315 who were deemed ineligible, making it difficult to estimate how many of them should have been eligible. We recognise that BMI and waist circumference per se are not accurate measures of adiposity and the cut-off points are arbitrary and that they do not differentiate between muscles and fat or take into account the full effect of height so that for example a short person’s cut-off points should probably be lower than for a taller person. This phenomenon may have affected participants "self-perceived obesity”. For some people self-perceived obesity might actually be a more accurate risk predictor than measured obesity. Other potential causes of miscalculation of FINDRISC such as misreporting family history, previous high glucose measurements, diet and exercise have not been addressed in this study.

## Conclusions

Risk-prediction tools can help to improve diagnosis of pre-diabetes in risk individuals, and therefore assist in preventing type 2 diabetes [26]. FINDRISC is a simple, inexpensive, non-invasive, validated and reliable tool designed to assess risk of type 2 diabetes quickly and efficiently in primary care settings [5,18]. FINDRISC has also been said to be the best available tool for use in clinical practice in Caucasian populations when comparing its accuracy, availability, practicability and cost to other commonly used risk-prediction tools [26]. The results from this study highlight this tool’s ability to capture individuals who are most at risk of diabetes.

As obesity is one of the major contributing risk factors for type 2 diabetes, the tools used to assess the level of overweight and obesity need to be carefully considered prior to recruitment into diabetes prevention programs. This is especially important as large-scale diabetes prevention programs are being implemented world-wide as a response to the rising burden of this costly obesity related disease.

## Competing interests

The authors declare no competing interests.

## Authors’ contribution

BP conceived and designed the study. EDJ and JAD were involved in acquisition of data. BP with AH analysed and interpreted the data. AH drafted the manuscript and was responsible for its revisions. EDJ and BP helped to draft the manuscript. JAD contributed to specific sections in the manuscript and was the chief investigator for the GGT DPP. All authors read and approved the final manuscript.

## Pre-publication history

The pre-publication history for this paper can be accessed here:

http://www.biomedcentral.com/1471-2458/12/510/prepub
